# Phase 1 trial of vamorolone, a first-in-class steroid, shows improvements in side effects via biomarkers bridged to clinical outcomes

**DOI:** 10.1016/j.steroids.2018.02.010

**Published:** 2018-03-08

**Authors:** Eric P. Hoffman, Valerie Riddle, Maxime A. Siegler, Daniel Dickerson, Miroslav Backonja, William G. Kramer, Kanneboyina Nagaraju, Heather Gordish-Dressman, Jesse M. Damsker, John M. McCall

**Affiliations:** a ReveraGen BioPharma, Rockville, MD, USA; b Department of Pharmaceutical Sciences, School of Pharmacy, Binghamton University – SUNY, Binghamton, NY, USA; c BioPharmAdvisors LLC, USA; d Department of Chemistry, Johns Hopkins University, 3400 N. Charles St., Baltimore, MD 21218 USA; e PRA Health Sciences, Lenexa, KS, USA; f Kramer Consulting LLC, USA; g Children’s National Medical Center, Washington, DC, USA

## Abstract

**Background:**

Glucocorticoid drugs are highly effective anti-inflammatory agents, but chronic use is associated with extensive pharmacodynamic safety concerns that have a considerable negative impact on patient quality of life.

**Purpose:**

Vamorolone (VBP15) is a first-in-class steroidal multi-functional drug that shows potent inhibition of pro-inflammatory NFkB pathways via high-affinity binding to the glucocorticoid receptor, high affinity antagonism for the mineralocorticoid receptor, and membrane stabilization properties. Pre-clinical data in multiple mouse models of inflammation showed retention of anti-inflammatory efficacy, but loss of most or all side effects.

**Experimental approach:**

We report first-in-human Phase 1 clinical trials (86 healthy adult males), with single ascending doses (0.1–20.0 mg/kg), and multiple ascending doses (1.0–20 mg/kg/day; 14 days treatment).

**Key results:**

Vamorolone was well-tolerated at all dose levels. Vamorolone showed pharmacokinetic and metabolism profiles similar to prednisone. Biomarker studies showed loss of side effects of traditional glucocorticoid drugs (bone fragility, metabolic disturbance, immune suppression). Suppression of the adrenal axis was 10-fold less than prednisone. The crystallographic structure of vamorolone was solved, and compared to prednisone and dexamethasone. There was overlap in structure, but differences in conformation at the C-ring where glucocorticoids interact with Asn564 of the glucocorticoid receptor. The predicted loss of Asn564 binding to vamorolone may underlie the loss of gene transcriptional activity.

**Conclusions and interpretations:**

Vamorolone is a dissociative steroid that retains high affinity binding and nuclear translocation of both glucocorticoid (agonist) and mineralocorticoid (antagonist) receptors, but does not show pharmacodynamic safety concerns of existing glucocorticoid drugs at up to 20 mg/kg/day.

## 1. Introduction

The largest class of steroidal hormones are those secreted by the adrenal glands targeting Type 1 nuclear hormone receptors, and include glucocorticoids (cortisol), sex steroids (estrogen, progesterone, testosterone), and mineralocorticoids (aldosterone). These hormones diffuse through cell membranes where they bind cytosolic Type 1 receptors, dimerize, translocate to the nucleus, and regulate gene transcription through binding of target gene regulatory elements. Some of the most widely prescribed drugs target these receptors or their synthetic pathways, including prednisone, statins, bisphosphonates, and many others.

The modulation of target gene transcription by ligand/receptor steroidal complexes is termed ‘genomic effects’ (also called transactivation) [1]. While most physiological responses to steroidal hormones and drugs have been attributed to their broad genomic effects, there is increasing recognition and interest in non-genomic effects where ligand/ receptor complexes interact with other proteins [2]. Research into the relative effects of genomic and non-genomic activities of steroidal hormones and drugs have been complicated by variable cross-reaction with multiple Type 1 receptors, the variable expression of receptors in specific cell types, and cell-specific ligand metabolism.

In the late 1940′s, cortisol, the adrenal steroid hormone responsible for circadian rhythms and stress response, was found to show strong anti-inflammatory effects in arthritis patients, leading to the Nobel Prize in 1950. Cortisol shows high affinity binding to both the glucocorticoid receptor (NR3C1) where ligand/receptor complexes dimerize, enter the cell nucleus, and show broad genomic effects in all cells and tissues.

Cortisol also shows high affinity binding (agonism) for the mineralocorticoid receptor (NR3C2). Aldosterone is the natural ligand for the mineralocorticoid receptor (NR3C2), where ligand-bound receptor alters sodium balance. Aldosterone typically circulates at much lower concentrations than cortisol, but aldosterone-responsive cells express high levels of 11β-Hydroxysteroid dehydrogenase (11BHSD) isoforms, leading to inactivation of cortisol to cortisone.

Synthetic variants of cortisol were developed as potent anti-inflammatory drugs showing less cross-reactivity for the mineralocorticoid receptor, and less binding to cortisol binding globulin (CBG) thus increasing bioavailability [3,4]. The drug class of glucocorticoids, including prednisone, deflazacort, dexamethasone, and fluticasone, have remained among the most prescribed drugs, with about 90 million prescriptions per year in the US (#3 after opioids and thyroxines). Indeed, they are considered front-line therapy for both acute and chronic inflammatory conditions.

The glucocorticoid class of hormones and drugs were initially named for their profound effects on glucose metabolism, where they induce insulin resistance within 8 h of an initial dose as low as 0.2 mg/kg [5,6]. Metabolic disturbance is one of many severe side effects typically seen with chronic use of glucocorticoid drugs [7]. Others include changes in bone metabolism leading to osteopenia, bone breaks, and avascular necrosis of femoral head [8], mood and psychiatric changes [9], skeletal muscle atrophy and weakness [10], hypertension and renal disturbances [11], and rapid and profound suppression of the HPA axis leading to broad endocrine disturbances. In children chronically treated with glucocorticoids, complaints of families include stunting of growth, delay of puberty, weight gain, mood disturbances and brittle bones [12].

Increasing evidence has suggested that anti-inflammatory efficacy of glucocorticoid drugs is predominantly via non-genomic effects, whereas side effects are via genomic effects [13]. Dissociative steroidal drugs dissociate genomic and non-genomic effects, with the goal of retaining anti-inflammatory efficacy while reducing the burden of side effects.

VBP-15 (generic name vamorolone) was developed as a dissociative steroid by synthesizing a series of compounds with a Δ9,11 steroidal scaffold, and testing of cell-based assays for retention of non-genomic anti-inflammatory activity (inhibition of TNF-induced NFκB activation; suppression of innate immunity pathways), and loss of genomic activity (GRE-mediated transcriptional reporters) [14,15]. Pre-clinical data has shown that vamorolone has potent binding to the glucocorticoid receptor and anti-inflammatory effects similar to traditional glucocorticoid drugs [15]. Testing of multiple mouse models of inflammatory states has shown efficacy similar to prednisolone, and dramatic improvements of side effect profiles, including loss of growth stunting and loss of bone fragility [15–19].

In addition to the dissociation of genomic (lost) and transrepression (retained) activities, vamorolone has changed additional sub-activities of traditional glucocorticoids. Cortisol and pharmacological glucocorticoids show high affinity for the mineralocorticoid receptor, similar to its physiological ligand, aldosterone. Glucocorticoids and aldosterone act as agonists for the mineralocorticoid receptor, and high circulating levels of pharmacological glucocorticoids result in salt imbalance and water weight gain (Cushingoid appearance) via the reninangiotensin system. We used in vitro reporter assays for multiple steroid hormone receptors, in both agonist and antagonist modes, and showed that vamorolone is a potent antagonist for the mineralocorticoid receptor, in contrast to the agonist activity of glucocorticoids (unpublished observations). A pharmacological antagonist for the mineralocorticoid receptor, epleronone, has shown efficacy for improving heart function in Duchenne muscular dystrophy, and many other cardiac disorders [20], suggesting that vamorolone may show benefit for cardiomyopathy in addition to its anti-inflammatory activity. Vamorolone can not be metabolized by 11β-Hydroxysteroid dehydrogenase (11BHSD) isoforms, and thus should show greater stability in aldosterone-responsive cells (e.g. not inactivated as cortisol and glucocorticoids are).

Another potential mechanism of action of glucocorticoids is via induction of mitotic synchrony in cells, which in turn reduces the proinflammatory state [21,22]. This mitotic synchronization activity may extend to regenerative synchronization and reduction of inflammation in tissues, including muscular dystrophy [23]. Vamorolone retains or improves this cell and tissue synchrony activity of glucocorticoids [23]. Finally, steroidal drugs may alter physicochemical properties of cell membrane systems when integrating and traversing lipid compartments. Vamorolone has been shown to impart stability to myogenic cell membranes, whereas prednisone de-stabilizes membranes [15]. This was anticipated because the C-ring of our delta 9,11 steroid has a lipophilic double bond rather than the hydrophilic hydroxy or ketone of glucocorticoids.

Here, we report the initial clinical experience with vamorolone in healthy adult volunteers. We assessed multiple side effects of traditional glucocorticoids using pharmacodynamic biomarkers, both acutely (after a single dose) and chronically (14 daily doses). All biomarkers studied were bridged to clinical safety outcomes, including adrenal suppression, insulin resistance, osteopenia, and immune suppression. The vamorolone program operates under a venture philanthropy model [24], working with stake holders and governments worldwide. This effectively de-risks the program, while also encouraging innovation.

## 2. Materials and methods

This clinical trial was a randomized, double-blind, placebo controlled single ascending (SAD) and multiple ascending (MAD) study to evaluate the safety, tolerability, and pharmacokinetics of vamorolone in health adult subjects. The study protocol and its amendments were reviewed and approved by an Institutional Review Board. It was conducted in accordance with the principles of the Declaration of Helsinki in place at the time of study conduct and in compliance with the International Council on Harmonisation (ICH) E6 Guideline for Good Clinical Practice.

### 2.1. Subject recruitment

After signing informed consent, a total of 286 subjects were screened, of whom 132 subjects met eligibility criteria and qualified for the study. Inclusion/exclusion criteria were those considered standard for studies of this type, including requirement for normal liver-related chemistries, lipase, and amylase. Of these 132 subjects, 86 were randomized, with 54 in the SAD part of the study and 32 in the MAD part. All 54 subjects in the SAD part and 32 in the MAD part received at least one dose of vamorolone or placebo and were included in the safety population. All 42 subjects who received vamorolone comprised the SAD PK analysis population.

In the SAD part, 12 subjects were assigned to placebo, and 6 subjects each were assigned to one of seven vamorolone dose groups. All 54 subjects in the SAD part completed dosing and all study procedures.

In the MAD part, 8 subjects were randomly assigned to each of four dose groups (2 placebo, 6 drug per dose group). Thirty-one of the 32 subjects in the MAD part completed the study. One subject, randomized to placebo, terminated the study early to seek care for exacerbation of a previously existing dental condition and did not complete dosing. Two additional subjects, one randomized to placebo and the other to 20 mg/kg vamorolone, discontinued dosing due to AEs but completed the study. The subject in the 20 mg/kg vamorolone dose group who did not complete dosing with vamorolone was excluded from the PK analysis; thus, the MAD PK population consisted of 23 subjects.

A total of 54 male subjects between 19 and 64 years of age, with a BMI range of 21.2 to 31.6 kg/m^2^, participated in the SAD part. The majority of subjects were white (28 subjects, 52%), followed by black (20 subjects, 37%). Five subjects (9%) were of Hispanic or Latino ethnicity.

A total of 32 male subjects between 18 and 45 years of age, with a BMI range of 19.6 to 30.3 kg/m^2^, and no females participated in the MAD part. The majority of subjects were white (16 subjects, 50%), followed by black (14 subjects, 44%). Two subjects (6%) were of Hispanic or Latino ethnicity.

Demographic characteristics were similar across dose groups in both the SAD and MAD parts of the study.

#### 2.1.1. Single ascending dose (SAD)

In the SAD part of the study, 7 cohorts were to be enrolled. Eight subjects were assigned to each of Cohorts 1 to 5 and 7 and received a single oral dose of vamorolone (planned as 0.1 mg/kg, 0.3 mg/kg, 1.0 mg/kg, 3.0 mg/kg, 8.0 mg/kg, and 20.0 mg/kg, respectively) or placebo under fasted conditions. Six subjects were assigned to Cohort 6 and were administered a single oral dose of 8.0 mg/kg vamorolone within 30 min of beginning a high fat/high calorie meal.

Subjects in Cohorts 1 through 5 and 7 were randomized to receive vamorolone or placebo in a 3:1 ratio (6 subjects received vamorolone, and 2 subjects received placebo) sequentially. In Cohort 1, 3 sentinel subjects were administered vamorolone or placebo in a 2:1 ratio. Following a review of sentinel safety and tolerability data (including reported adverse events [AEs], clinical laboratory tests, vital signs, and electrocardiograms [ECGs]), the remaining 5 subjects were randomized to receive vamorolone or placebo in a 4:1 ratio and dosed no earlier than 48 h after sentinel dosing. In Cohorts 2 to 5, and 7, subjects were administered vamorolone or placebo in a 3:1 ratio. Once Cohort 5 was completed and safety data had been reviewed, subjects in Cohort 6 were administered a single oral dose of 8.0 mg/kg vamorolone within 30 min of beginning a standard high fat/high calorie meal.

After completion of all scheduled safety follow-up visits for a given cohort, a Safety Review Committee (SRC), comprised of the Principal Investigator (PI), the Sponsor’s Medical Monitor, and the Sponsor’s pharmacokineticist, reviewed all available PK and safety data (AEs, physical examination findings, ECGs, vital signs, and clinical laboratory tests) in a blinded manner for the completed cohort prior to making a decision to escalate to the next higher dose level. Data unblinding did not occur until after database lock.

Dose escalation was to be halted if any of the following occurred:

1. The presence of a serious adverse event (SAE) in 1 subject within a cohort who received vamorolone considered by the PI to be related to the investigational product (IP).
2. The presence of the same system (eg, gastrointestinal, cardiac) Grade 3 or greater AE, considered related to IP, in 3 or more subjects in the same cohort, provided the affected subjects received vamorolone.
3. The presence of an elevation in alanine aminotransferase (ALT) of greater than or equal to 3 times the upper limit of normal (ULN) in conjunction with an elevation in total bilirubin of greater than or equal to 2 times the ULN in 1 subject within a cohort if considered IP-related by the PI, the subject received vamorolone, and no alternative explanation could be identified.
4. Any other clinical observation that the SRC or PI considered a safety issue such that the higher doses of vamorolone should not be administered to subsequent subjects.

#### 2.1.2. Multiple ascending dose (MAD)

The MAD part of the study was initiated after a review of safety and PK data from SAD cohorts 1 to 6. In the MAD part, 4 cohorts containing 8 subjects each were randomized to receive vamorolone or placebo, in a 3:1 ratio, once daily (QD) for 14 days. The duration of dosing in the MAD part was determined based on the PK data from the SAD part. In each cohort, 3 sentinel subjects were administered vamorolone or placebo, in a 2:1 ratio. Once the sentinel group demonstrated that the dose was generally safe and well tolerated, based on a review of sentinel safety and tolerability data (including reported AEs, clinical laboratory tests, vital signs, and ECGs) after QD dosing for 4 days, dosing in the sentinel group continued, and the remaining 5 subjects were randomized to receive vamorolone or placebo in a 4:1 ratio and dosed QD for 14 days.

The planned dose levels for the MAD part were 1.0 mg/kg (Cohort 1), 3.0 mg/kg (Cohort 2), 9.0 mg/kg (Cohort 3), and 20.0 mg/kg (Cohort 4) or until the maximum tolerated dose (MTD) was identified.

After completion of all scheduled safety follow-up visits for a given cohort, the SRC reviewed all available PK and safety data (AEs, physical examination findings, ECGs, vital signs, and clinical laboratory tests) in a blinded manner for the completed cohort prior to making a decision to escalate to the next higher dose level. Data unblinding did not occur until after database lock.

Dose escalation in the MAD part was to proceed according to protocol until the MTD was identified. The dose escalation stopping criteria, as listed above in the SAD section, comprised the halting rules for the MAD part. If any of these criteria were met during the 14-day dosing duration in the MAD part, dosing in that cohort was to be halted and no further dose escalation was to occur. If AEs/SAEs occurred more frequently in subjects treated with vamorolone compared to subjects treated with placebo, the SRC was to determine if dose escalation could continue. If dose escalation was terminated, the remaining cohorts could be enrolled to evaluate the safety and PK effects of vamorolone for dose levels between the previously tolerated dose and the dose level at which dose escalation was terminated. Once safety data from additional cohort(s) was assessed, the MTD was defined as the dose level below the dose at which the SRC determined there was an unacceptable risk to subjects.

Safety, including criteria for stopping early, was continuously assessed by the Medical Monitor. Subjects in MAD cohorts 1 to 4 were asked permission to keep any remaining specimens for possible use in future research studies, such as testing for biomarkers, but no human genetic testing was performed on these samples.

An MTD was not reached during MAD dosing, and all 4 planned doses were administered.

### 2.2. Safety

In the SAD part, overall, six subjects (11%) administered vamorolone experienced a total of 10 treatment-emergent adverse events (TEAEs); no subject in the placebo group experienced any TEAEs. There was no dose-related trend in the incidence or severity of TEAEs; the dose group with the highest number of subjects reporting TEAEs was the 0.1 mg/kg dose group (2 subjects, 33%), and the highest number of TEAEs (3 events) was reported by 1 subject in the 1.0 mg/kg vamorolone dose group. In the 0.3 and 3.0 mg/kg vamorolone fasted and the 8.0 mg/kg fed dose groups, 1 subject per group (17%) experienced TEAEs, and no subjects in the 8.0 mg/kg and 20 mg/kg, fasted, dose groups experienced any TEAEs. The most common TEAEs were dizziness and headache, each reported by 2 subjects overall (4%); all other TEAEs were reported by only 1 subject (2%) and included ear pain, nausea, non-cardiac chest pain, and blood bilirubin increased. Three subjects (6%) had TEAEs that were considered possibly related to treatment. Possibly related TEAEs included nausea (1 subject, 2%), dizziness (2 subjects, 4%), and headache (2 subjects, 4%). One subject (2%) had a moderate TEAE of blood bilirubin increased, which was considered unrelated to study drug. All other TEAEs were mild in severity.

In the MAD part, overall, a total of 6 subjects (19%) administered vamorolone or placebo experienced a total of 10 TEAEs: 2 subjects each in the 1.0 mg/kg vamorolone, 20 mg/kg vamorolone, and placebo groups, and none in the 3.0 mg/kg and 9.0 mg/kg dose groups. There was no dose-related trend in the incidence or severity of TEAEs. The most common TEAE was headache (2 subjects, 6%); all other TEAEs occurred in only 1 subject (3%) per group, and included nausea, toothache, vomiting, ALT increased, hepatic enzyme increased, anthralgia, dizziness, and syncope. TEAEs were considered possibly related in 2 subjects (6%) and remotely related in 1 subject (3%). Possibly related AEs were ALT increased and hepatic enzyme increased, occurring in 1 subject in the 20 mg/kg vamorolone and placebo groups, respectively. The remotely related TEAEs were dizziness and syncope, both occurring in the same subject (1.0 mg/kg vamorolone). All TEAEs were mild in severity.

With the exception of the AEs related to hepatic enzymes, there were no other meaningful changes in clinical laboratory parameters

### 2.3. Biomarker methods

All biomarkers were analyzed using standard immunoassays by PRA.

### 2.4. Single crystal X-ray crystallography

All reflection intensities were measured at 110(2) K using a SuperNova diffractometer (equipped with Atlas detector) with Cu Kα radiation (λ = 1.54178 Å) under the program CrysAlisPro (Version 1.171.36.32 Agilent Technologies, 2013). The same program was used to refine the cell dimensions and for data reduction. The structure was solved with computer programs *CrysAlis PRO*, Agilent Technologies, Version 1.171.36.32 (release 02-08-2013 CrysAlis171.NET) (compiled Aug 2, 2013, 16:46:58), *SHELXS2014*/7, *SHELXL2014*/7, and *SHELXTL* v6.10 [25].

Analytical numeric absorption correction using a multifaceted crystal model was applied using CrysAlisPro. The temperature of the data collection was controlled using the system Cryojet (manufactured by Oxford Instruments). The H atoms were placed at calculated positions using the instructions AFIX 13, AFIX 23, AFIX 43, AFIX 137 or AFIX 147 with isotropic displacement parameters having values 1.2 or 1.5 *U*eq of the attached C or O atoms.

The structural coordinates of the X-ray crystal structure of vamorolone have been deposited in the Cambridge Crystallographic Data Centre, deposition number CCDC 1557034.

## 3. Results

### 3.1. Pharmacokinetics: Single ascending dose

The initial starting dose for the vamorolone SAD study was chosen based on 10% of the NOAEL in the most sensitive species (mouse) (0.1 mg/kg). Doses were then escalated by 3-fold increments for 6 dose groups (0.1, 0.3, 1.0, 3.0, 9.0, 20.0 mg/kg). Eight adult male volunteers were studied in each dose group (6 drug-treated, 2 placebo).

**Fig. 1:**
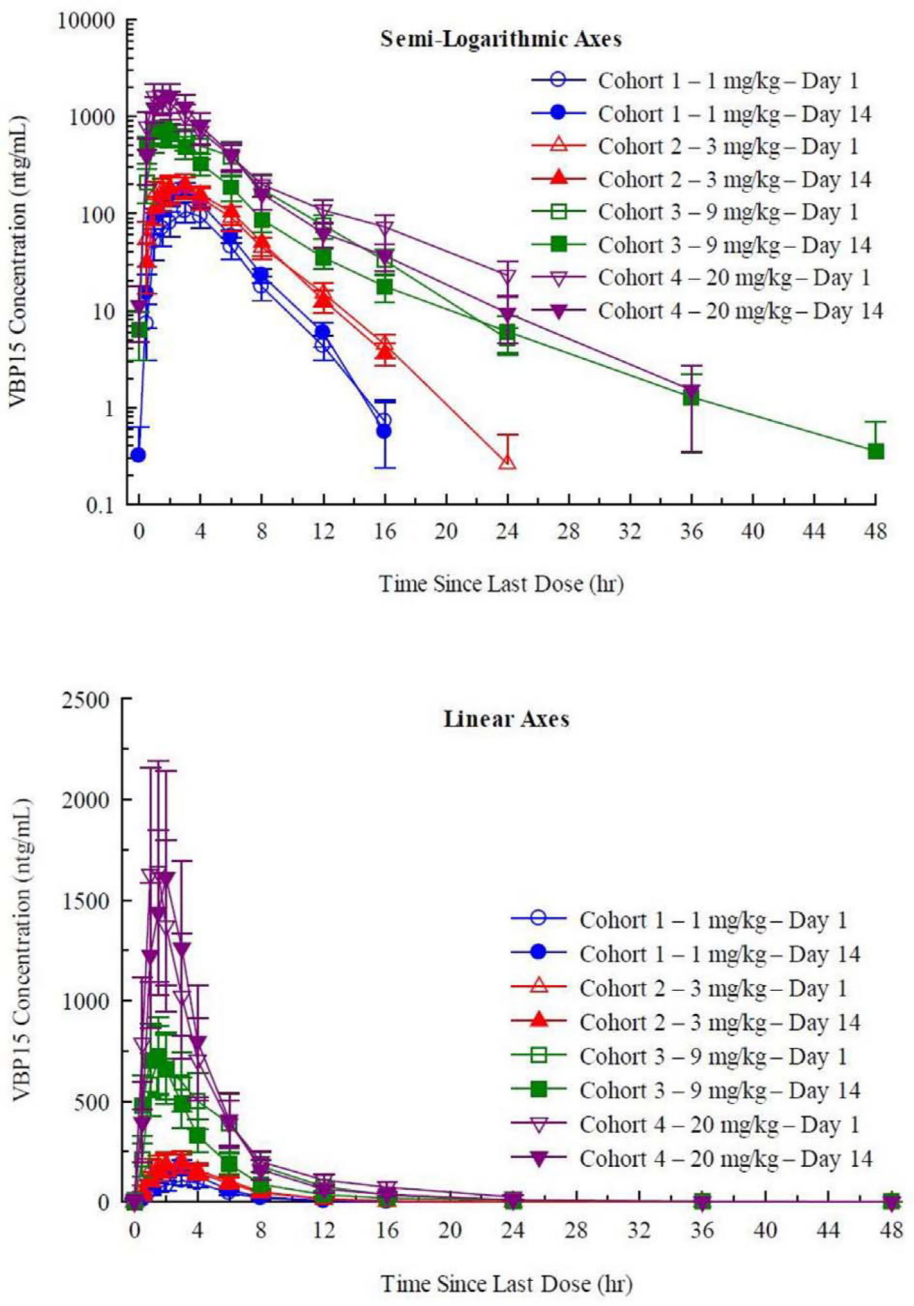
Pharmacokinetic analysis of vamorolone single ascending dose trial (SAD). Shown are arithmetic mean ± standard error plasma concentrations of vamorolone after oral administration of single 0.1, 0.3, 1, 3, 8, and 20 mg/kg doses to healthy subjects under fasted conditions — linear (top panel) and semi-logarithmic (bottom panel) axes. The data shows strong dose proportionality, short half-life (~ 3 h), and relatively little interindividual variability.

There were dose-related increases in the mean vamorolone plasma concentrations and dose-related pharmacokinetic (PK) parameter values after administration of single 0.1 mg/kg to 20 mg/kg doses (Fig. 1, Table 1). Log plots of the individual subject Cmax and AUC(inf) were linear. Although the slope for Cmax was < 1.0, that for AUC(inf) was close to 1.0 and indicates a linear or dose-proportional increase in exposure. The geometric mean values for CL/F ranged from 1.76 to 2.31 L/hr/kg or 142 to 196 L/hr with no apparent dependence on dose, providing further evidence of linear PK (Table 1). The geometric mean t½ range was somewhat longer at the higher doses, due primarily to vamorolone being quantifiable in plasma for a longer duration in the higher dose groups (e.g. above limits of detection) (Table 1).

**Fig. 2:**
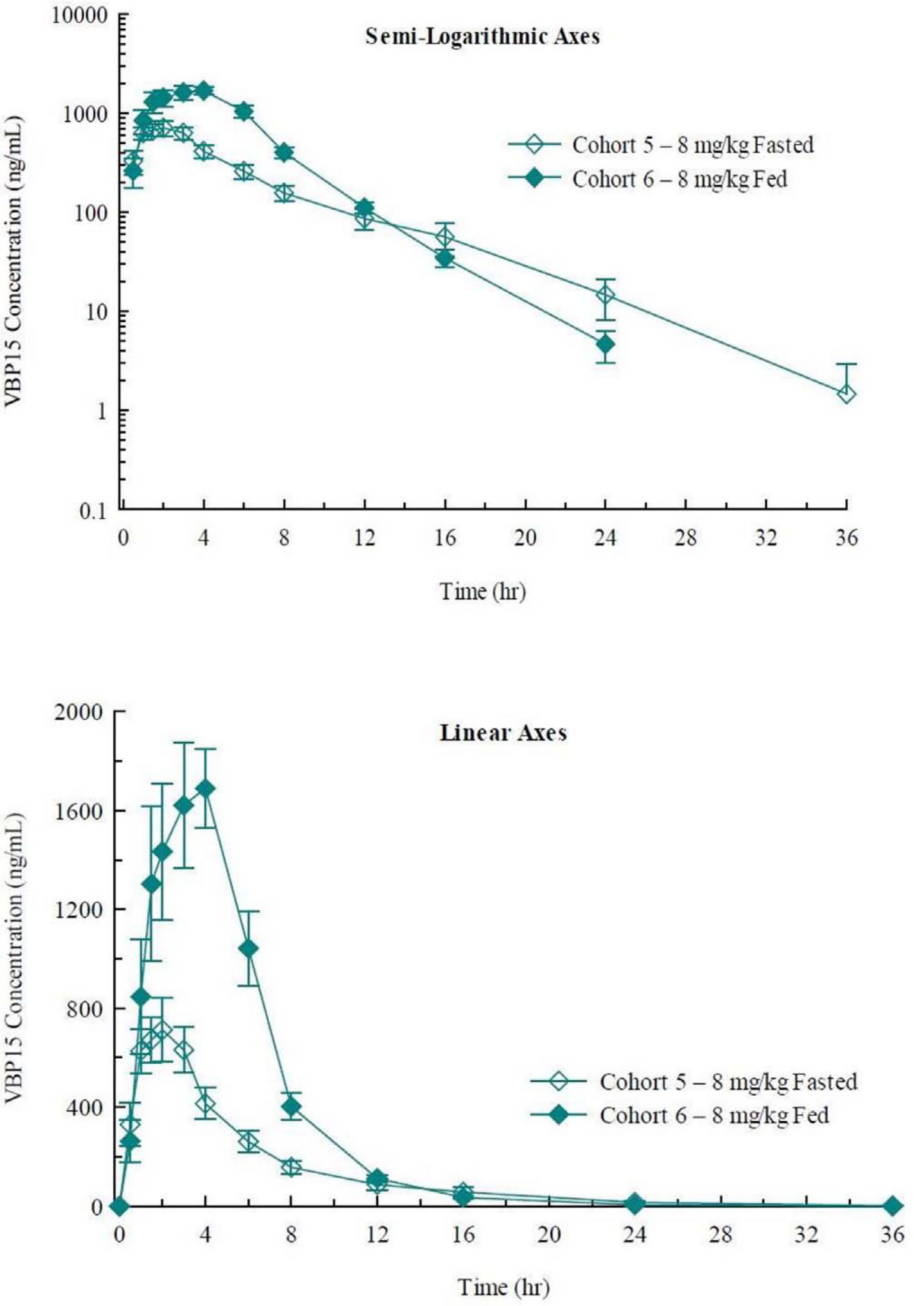
Pharmacokinetic analysis of vamorolone for food effect. Shown are arithmetic mean ± standard error plasma concentrations of vamorolone after oral administration of single 8 mg/kg doses to healthy subjects under fed and fasted conditions — linear (top panel) and semi-logarithmic (bottom panel) axes. This shows a significant food effect, with a 250% increase in bioavailability with a 40 g fat meal.

Administration of vamorolone with a high calorie/high fat meal (40 g fat) resulted in an increase in the mean plasma concentrations (Fig. 2). There was a 2.5-fold increase in Cmax, AUC(0-t), and AUC(inf) and an ~2-fold increase in Tmax under fed conditions (Table 2). This indicates that there is an increase in the extent of absorption and decrease in the rate of absorption when vamorolone is administered with this meal.

### 3.2. Pharmacokinetics: Multiple ascending dose (MAD)

**Fig. 3:**
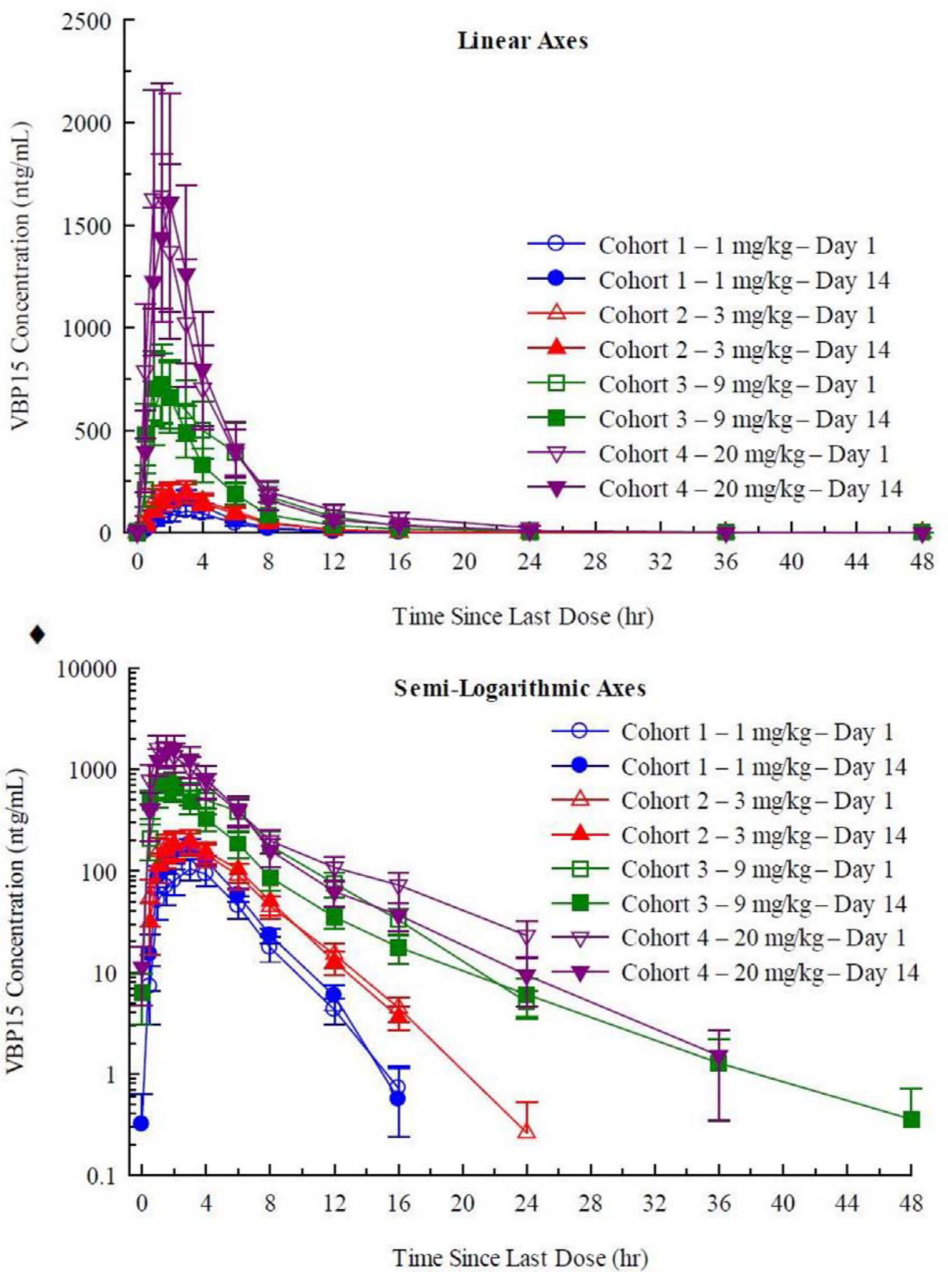
Pharmacokinetic analysis of multiple ascending dose (MAD) trial. Shown are arithmetic mean ± standard error plasma concentrations of vamorolone on Days 1 and 14 during oral administration of 1, 3, 9, and 20 mg/kg doses once daily for 14 days to healthy subjects under fasted conditions (linear axes [top panel] and semi logarithmic axes [bottom panel]). Day 1 and Day 14 PK data were superimposable for each dose group, showing that there was little or no drug accumulation between daily doses, consistent with the short half-life of vamorolone. Bioavailability was not significantly changed with 14 days of daily dosing.

Daily doses of vamorolone (1.0, 3.0, 9.0 and 20.0 mg/kg/day) were given in the morning in a fasted state, daily for 14 days, and PK determined on day 1 (after single dose) and day 14 (after the 14th dose). Consistent with the SAD, mean plasma vamorolone concentrations and dose-related PK parameters increased in a dose related manner (Fig. 3). Log-log plots of the individual subject Cmax and AUC (inf) were linear. Although the slopes ranged from 0.827 to 0.898, the deviation from 1.0 may be a consequence of between-subject variability and not indicative of a less than dose-proportional increase in exposure.

Consistent with the t½ relative to the 24-hour dosing frequency, there was essentially no drug accumulation between doses, as evidenced by essentially superimposable plasma concentrations of day 1 and day 14 PK curves (Fig. 3). Within the variability intrinsic to small groups of subjects (n = 6 per dose group), the geometric mean values for Cmax and AUC (0–24) on Days 1 and 14 were not significantly different. Consequently, “steady-state” is the same as a single dose for daily dosing with vamorolone.

The geometric mean values for CL/F ranged from 1.42 to 2.02 L/hr/kg (105 to 168 L/hr) on Day 1 and from 1.26 to 2.15 L/hr/kg (91.7 to 167 L/hr) on Day 14 with no apparent dependence on dose, providing further evidence of linear PK. As with the SAD, the geometric mean t½ range was somewhat longer at the higher doses, due primarily to vamorolone being quantifiable in plasma for a longer duration. There were no apparent relationships between CL/F and body size, either as body weight or BMI. This suggests that vamorolone should be administered as a fixed rather than body size-based dose in adults.

### 3.3. Pharmacodynamic safety biomarkers bridged to clinical outcomes

Four safety concerns of traditional glucocorticoid drugs were measured using serum biomarkers; adrenal suppression, bone turnover, insulin resistance, and immune suppression. Biomarkers utilized to monitor these four safety concerns were each bridged to long-term morbidities and changes in quality of life.

#### 3.3.1. Adrenal suppression

Adrenal suppression is the consequence of a negative feedback loop between high circulating cortisol and pharmacological glucocorticoids and adrenal function, leading to loss of responsiveness of the adrenal cortex to ACTH. Adrenal suppression can be seen both acutely (hours after a single dose of pharmacological glucocorticoids), and chronically. Chronic treatment with glucocorticoids leads to chronic deficiencies in multiple steroidal hormones, including cortisol, testosterone, 17-hydroxyprogesterone, corticosterone, and 11-deoxycortisol [26]. The resulting hormonal imbalance puts patients at increased risk for a series of safety concerns. One is adrenal crisis (inability to mount a cortisol stress response after trauma, severe illness, or surgery, with ensuring risk for shock and death). Other safety concerns in children include delay of puberty and growth stunting. Adrenal suppression can be measured with first-in-morning serum cortisol, reflective of the endogenous diurnal cortisol peak pre-awakening. Measurement of first-in-morning cortisol 24 h after the last drug dose indicates whether the drug has suppressed the normal diurnal cortisol response. First-in-morning cortisol levels below 3.6 µg/dL suggest that the adrenal cortex is not able to mount a diurnal cortisol response, and adrenal function is suppressed. Prednisone causes adrenal suppression at doses of 0.1–0.2 mg/kg, both acutely (24 h after first drug dose) and chronically (7 days of daily drug dosing) [5,6].

**Fig. 4:**
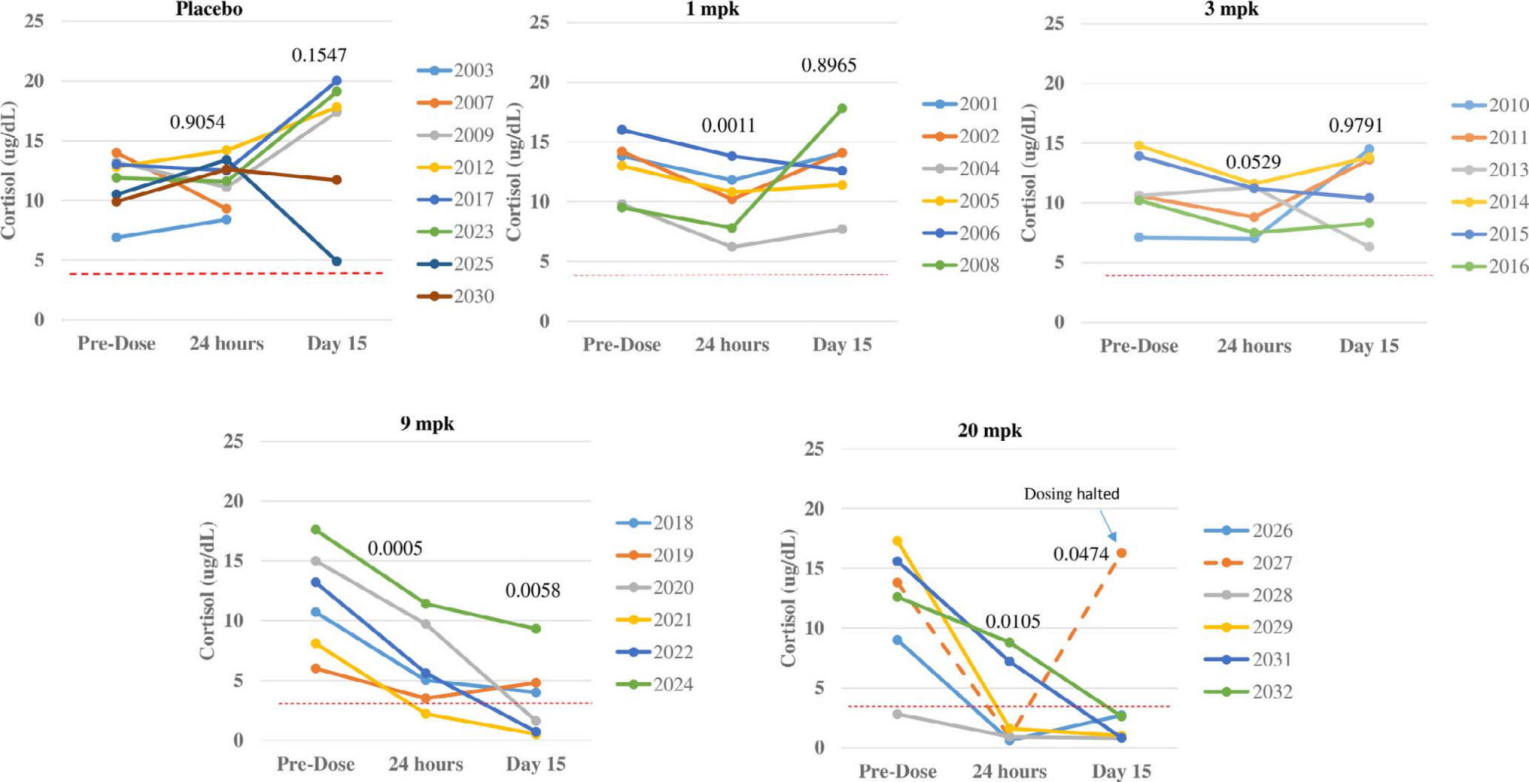
First-in-morning serum cortisol shows evidence for adrenal suppression at high doses. First-in-morning cortisol was measured in Phase 1 volunteers as in-patients in the Phase 1 clinical trial unit. Cortisol and glucocorticoid drugs cause rapid suppression of the adrenal axis, with marked reductions in serum cortisol within 8 hrs of the first 0.25 mg/kg dose (*5,6*). Adrenal suppression is often defined as inability to mount a morning cortisol response (diurnal fluctuations), and a diagnostic threshold for this is when first-in-morning cortisols fall to < 100 nmol/L (< 3.6 Âµg/dL). Morning cortisol levels of adult volunteers treated with 1.0 and 3.0 mg/kg/day vamorolone showed successful mounting of a morning cortisol response, with patterns similar to placebo. Subjects receiving 9.0 mg/kg/day showed a mild decrease at 24 hrs after the first dose (paired T = 0.0005), with one of six subjects falling 100 nmol/L at 24 h, and three of six (50%) after two weeks of daily dosing. Subjects receiving 20 mg/kg/day showed stronger adrenal suppression, with four of six falling below the 100 nmol/L threshold at 24 h after the first dose, and 100% (five of five) after two weeks of daily dosing. One subject had dosing stopped after 10 days due to mild elevations of liver enzyme (dashed red line). The adrenal function of this subject returned to baseline levels at 5 days after cessation of dosing.

First in morning serum cortisol levels were measured in all Phase 1 MAD subjects, at baseline prior to first administration of vamorolone or placebo, 24 h after the first dose (acute suppression), and 24 h after 14 days of daily dosing (chronic suppression) (Fig. 4). The two placebo subjects from each dose group were combined into the same panel. No evidence of adrenal suppression was seen in the 1.0 or 3.0 mg/kg/day dose groups. At 9.0 mg/kg/day, there was a decline in morning cortisol in all subjects, with 1 subject below the 3.6 µg/dL after first dose, and 3 of 6 subjects after two weeks of daily dosing. At 20.0 mg/kg/day, 4 of 6 showed adrenal suppression after a single dose, and all subjects (5/5) after two weeks of dosing. One subject stopped dosing due to mild elevation of liver enzymes, and his morning cortisol returned to normal levels within a few days of cessation of dosing (Fig. 4). Direct head-to-head comparisons to prednisone-treated subjects were not done in this Phase 1 trial, but approximate comparisons can be done to published literature [6]. Both Fleishaker et al. [6] and we tested serum cortisol 24 h after the first dose of drug. Fleishaker et al. studied doses of 2.5, 5, 10, 20 mg, 40 mg, and 60 mg, with an average weight of subjects of 80 kg. Converting this to mg/kg, the prednisone groups were 0.035, 0.07, 0.15, 0.25 mg/kg, 0.5 mg/kg, and 0.75 mg/kg. At 24 h after the initial dose, reductions in morning cortisol 24 h later were seen in the 20 mg (0.25 mg/kg) (9 µg/dL cortisol), 40 mg (0.5 mg/kg) (7 µg/dL cortisol), and 60 mg (0.75 mg/kg) (6 µg/dL cortisol). For vamorolone, the dose groups showing decreases in morning cortisol 24 h after the initial dose were 3 mg/kg (9 µg/dL cortisol), 9 mg/kg (6 µg/dL cortisol), and 20 mg/kg (4 µg/dL cortisol). Aligning the dose/response of prednisone vs. vamorolone, 0.25 mg/kg prednisone was equal to 3.0 mg/kg vamorolone (9 µg/dL cortisol), and 0.75 mg/kg prednisone equal to 9 mg/kg vamorolone (6 µg/dL cortisol). Thus, for acute measures of adrenal suppression 24 h after the first dose, there was approximately a 10-fold difference in potency (prednisone > vamorolone). Two week prednisone treatment data for morning cortisol was not published.

#### 3.3.2. Insulin resistance

Glucocorticoids were originally named as such due to their potent and acute effects on glucose metabolism, where liver and muscle pathways for insulin signaling are rapidly and profoundly affected, creating a state of insulin resistance. Prednisone has been shown to lead to detectable insulin resistance within five hours of a single 0.2 mg/kg dose [5,27]. Hyperglycemia and hyperinsulinemia are also seen 24 h after a single 2.0 mg/kg dose of prednisone [28]. Fasting insulin and glucose levels were assessed in all dose groups in the vamorolone Phase 1 SAD and MAD. Levels did not differ from placebo for any dose group at any time point and were within normal ranges (Supplemental Fig. 1; Supplemental Tables 1 and 2). This indicates that vamorolone has lost the metabolic side effect of insulin resistance through 20 mg/kg/day.

#### 3.3.3. Bone turnover

**Fig. 5:**
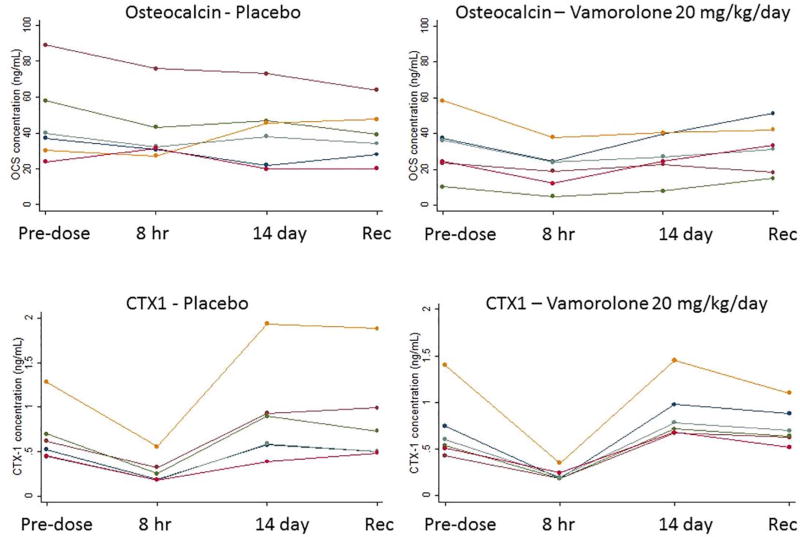
Vamorolone shows no changes in bone turnover biomarkers. Osteocalcin (biomarker for bone formation), and CTX1 (carboxy-terminal cross-linked telopeptides of type 1 collagen; biomarker for bone resorption). Data is shown for placebo and 20 mg/kg/day dose groups (complete data in Supplemental Tables). Prednisone shows significant decreases in osteocalcin levels at 8 h after a single 0.2 mg/kg dose, and after seven days of daily dosing (Kauh et al. 2012). Vamorolone did not show significant decreases of osteocalcin at any dose. In Kauh et al., a biomarker for bone resorption, NTX (amino-terminal cross-linked telopeptides of type 1 collagen), showed significant increases after seven days of treatment with prednisone at 0.25 mg/kg/day, whereas vamorolone did not shown any changes of CTX1 (a similar biomarker) through the highest dose tested (20 mg/kg/day). This data suggests that vamorolone does show the safety concern of bone fragility, and is consistent with pre-clinical data on vamorolone, consistent with mouse data (Heier et al. 2013).

Pharmacological glucocorticoids cause bone side effects including osteopenia and bone fragility [29]. Bone turnover markers bridged to later bone morbidities include osteocalcin for bone formation, and CTX1 (C-terminal telopeptide of collagen 1) for bone absorption. These biomarkers predict phenotypes of osteopenia and used in clinical trials to monitor bone side effects of treatments [30]. A single dose of prednisone as low as 0.2 mg/kg leads to rapid reductions in osteocalcin [5] (Supplemental Fig. 2). In the vamorolone Phase 1 trial, serum osteocalcin levels were assayed prior to dosing (Day 1 predose), 8 h after initial dose (Day 1), pre-dose on day 14 of daily dosing, 8 h after the day 14 (last) dose, and during recovery days 15 and 17. Osteocalcin and CTX1 showed no significant changes in any vamorolone dose group compared to placebo (Fig. 5; Supplemental Tables 3 and 4). This suggests that vamorolone has lost the side effect of bone turnover and osteopenia, consistent with published pre-clinical data in mouse models showing no osteopenia or stunting of growth [15]. CTX1 was measured in the same samples and showed no significant changes at any dose level of vamorolone.

#### 3.3.4. Immune suppression

A single dose of pharmacological glucocorticoids results in rapid changes in peripheral blood white cell counts, with increased neutrophils, and decreased lymphocytes [6]. Increases of neutrophils are from release from peripheral tissues, whereas loss of lymphocytes is due to induction of apoptosis [3,31]. Differential white blood cell counts were studied in all Phase 1 MAD subjects. No significant changes in any white blood cell count were seen in any dose group (Supplemental Fig. 3; Supplemental Tables 58). This suggests that vamorolone has lost the safety concern of immune suppression.

### 3.4. Clinical safety findings in the Phase 1 trials

There were no severe adverse events observed in any SAD or MAD dose group. One subject in the 20 mg/kg/day MAD cohort showed mild elevations of liver enzymes that were thought to be drug related, and dosing was halted. This subject had mild elevations of bilirubin prior to drug dosing, suggesting there may have been a pre-existing liver condition that may have been exacerbated by high doses of vamorolone, as has been reported for prednisone. The human NOAEL was determined by the Phase 1 study director to be 20 mg/kg/day, the highest dose tested. The adrenal suppression seen at the highest vamorolone dose groups was viewed as a reversible pharmacological safety finding by the study director, and not classified as an adverse event.

### 3.5. X-ray structure determination for vamorolone and comparison to glucocorticoids

The structure of vamorolone was solved in the non-centrosymmetric spacegroup *P*2_1_2_1_2_1_ with Z’ = 3 (i.e., there are three crystallographically independent molecules in the asymmetric unit). The structure is ordered. The absolute configuration was established by anomalous-dispersion effects in diffraction measurements on the crystal, and the Flack and Hooft parameters both refine to −0.02(3). The correct chirality on C8/C10/C13/C14/C16/C17 is S/R/S/S/R/R. All relevant crystallographic data are provided in Supplemental Table 9.

**Fig. 6:**
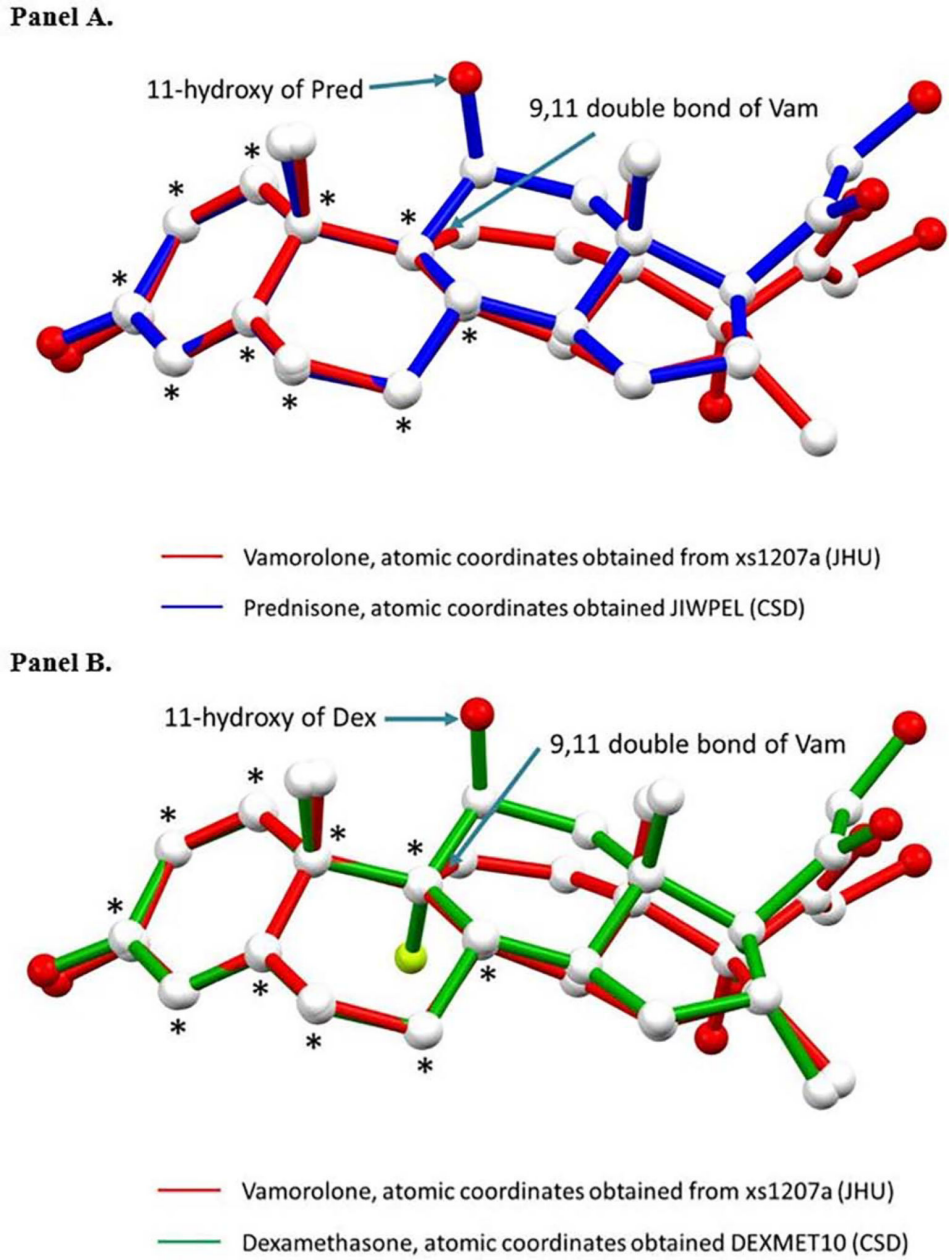
Comparison of the experimentally determined crystal structures of vamorolone and glucocorticoids (prednisone, dexamethasone). Panel A. Structure Overlay of Vamorolone (Vam, red) and Prednisone (Pred, blue). Overlay was calculated based on the fit of the 10C atoms marked as *, r.m.s = 0.0627. Panel B. Structure Overlay of Vamorolone (Vam, red) and Dexamethasone (Dex, green). Overlay was calculated based on the fit of the 10C atoms marked as *, r.m.s = 0.0622.

The crystal structure of vamorolone was overlaid to previously reported crystal structures of prednisone and dexamethasone [32] (Fig. 6). While there is considerable overlap in structures with both glucocorticoids, the principal structural and molecular difference results from vamorolone’s double bond that orients the C-ring differently than the C-rings of the two comparison glucocorticoids.

## 4. Discussion

We studied the response of 86 adult volunteers to vamorolone over a broad dose range (SAD 0.1–20.0 mg/kg; MAD 1.0–20.0 mg/kg/day 14-day treatment period). For clinical safety, no severe adverse events were observed, and the NOAEL in humans was defined as the highest dose tested (20.0 mg/kg/day). The pharmacokinetics of vamorolone was well-behaved, with excellent dose proportionality, and little variation between subjects (Figs. 1 and 3). Vamorolone is a steroid with very poor solubility in aqueous solutions, similar to traditional glucocorticoids and cortisol. Vamorolone bioavailability showed a pronounced food effect, where ingestion of a 40 g fat meal increased bioavailability by 250% (Fig. 2). Despite the poor solubility, oral delivery with a suspension formulation (4% vamorolone by weight) showed excellent bioavailability, with peak serum concentrations at ~3 h. Both the bioavailability and half-life of vamorolone (~3 h) were similar to other glucocorticoids. Most glucocorticoids are prescribed as a once-daily regimen, and this is effectively a pulsed dose with little or no drug accumulation between doses; this was observed in the MAD study, where daily dosing showed little or no drug accumulation between doses (Fig. 2). We are currently carrying out Phase 2a clinical studies in 4 to < 7 years Duchenne muscular dystrophy children using a daily dosing regimen (pulsed drug exposure) despite the lack of achieving steady state. This decision was based upon the desire to benchmark against daily prednisone and deflazacort in DMD, as well as increasing evidence of a mitotic and regenerative re-synchrony activity of both glucocorticoids and vamorolone that may benefit from a pulsed drug exposure [22–24].

Vamorolone is being developed as a replacement for glucocorticoid treatment of Duchenne muscular dystrophy and other chronic inflammatory conditions. Vamorolone has shown efficacy similar or superior to prednisone in mouse models of muscular dystrophy [15,23], lung disease [16,19,24], inflammatory bowel disease [18], and multiple sclerosis [17]. These *in vivo* pre-clinical studies also showed improvement of bone safety profiles of vamorolone when benchmarked against prednisone tested in parallel, including loss of stunting of growth and osteopenia [15,17,18]. Measures of immune suppression were studied both *in vitro* and *in vivo*, where CD4 cells declined with prednisone, but not vamorolone [15]. It is important to note that the potent immunosuppression effects of glucocorticoids are considered an aspect of efficacy in certain clinical indications (leukemias, and humoral autoimmunity disorders), but is considered a safety concern when prescribed in most chronic inflammatory conditions such as Duchenne muscular dystrophy, arthritis, asthma and others. Adrenal suppression was studied using *in vitro* assays in multiple cells types, and vamorolone consistently showed about ~1% of the adrenal suppressive activity of traditional glucocorticoids [14,15,19]. Safety concerns of metabolic derangement (insulin resistance) have not been studied in pre-clinical models to date.

The first-in-human safety data obtained for vamorolone in adult volunteers agrees with and extends the pre-clinical data. Adrenal suppression by vamorolone was monitored by biomarker studies of morning cortisol (diurnal response of the HPA axis) in the MAD, both 24 h after the first dose, and 24 h after 14 days of dosing. In dose ranging studies of prednisone in adult volunteers (2.5 mg–60 mg), evidence for blunting of the diurnal cortisol response 24 h after the first prednisone dose was seen in the 20 mg group (0.25 mg/kg) (mean = 9 µg/dL cortisol), 40 mg group (0.5 mg/kg) (mean = 7 µg/dL cortisol), and 60 mg group (0.75 mg/kg) (mean = 9 µg/dL cortisol). Comparison to vamorolone data 24 h after the initial dose (Fig. 4), showed about 10-fold less suppression of the diurnal cortisol response, with vamorolone responses at 3.0 mg/kg (mean =9 µg/dL cortisol), 9 mg/kg (mean = 6 µg/dL cortisol) and 20 mg/kg (mean = 4 µg/dL cortisol). Using a benchmark of morning cortisol levels < 3.6 µg/dL as evidence of inability of the HPA axis to mount a diurnal cortisol response (potential adrenal insufficiency), vamorolone showed no evidence of acute (single dose) or chronic (14 doses) adrenal suppression in the 1.0 and 3.0 mg/kg/day MAD groups (Fig. 4). Volunteers treated with 9 mg/kg/day and 20 mg/kg/day showed evidence of adrenal suppression, with the failure to mount a diurnal cortisol response for a subset of subjects 24 hrs after the first dose (1 of 6 subjects for 9 mg/kg/day; 4 of 6 for 20 mg/kg/day), and after 14 days of daily dosing (3 of 6 subjects for 9 mg/kg/day; 5 of 5 for 20 mg/kg/day). One subject in the 20 mg/kg/day showed mild elevations of liver enzymes that were believed to be drug-related, and this subject’s dosing was stopped at 10 days; his adrenal function returned to baseline levels when tested five days later (Fig. 4). Thus, both pre-clinical (mouse) and human Phase 1 data suggest that vamorolone shows considerably less adrenal suppression and associated safety concerns compared to glucocorticoids.

Consistent with bone findings in pre-clinical models, where prednisone (5 mg/kg/day) induced severe stunting of growth and osteopenia, yet vamorolone (30 mg/kg/day) did not [15], we found no alterations of bone turnover markers at any dose of vamorolone in the adult volunteers (Fig. 5). Bone morbidity due to chronic treatment of glucocorticoids are among the chief safety concerns of families and physicians [33,34], and prednisone causes both acute and chronic changes of bone turnover markers at doses of 0.2 mg/kg/day [5,6]. The lack of any changes in bone turnover markers in vamorolone-treated volunteers through 20 mg/kg/day is particularly promising regarding potential improved safety of this dissociative steroid.

Metabolic derangement and immunosuppression are key safety concerns of glucocorticoid drugs. Metabolic derangement is pervasive, with rapid and profound induction of insulin resistance, weight gain, and Cushingoid appearance. Studies of insulin resistance have not been reported in pre-clinical studies of vamorolone. Here, we found no evidence of drug-induced hyperglycemia at any dose of vamorolone, suggesting that insulin resistance was not induced by vamorolone. Likewise, we found no evidence of loss of blood lymphocytes in any vamorolone treated subject, consistent with lack of immunosuppression in pre-clinical studies [15].

The crystal structures of vamorolone, dexamethasone, and prednisolone share some similarities in conformation (Fig. 6). This is not surprising given the strong similarities in the A, the B and the D rings. The greatest difference lies in the C ring in which vamorolone has a C═C double bond between carbons 9 and 11 whereas in the more conventional glucocorticoids position 9 is a methylene and position 11 sports a 11-beta hydroxy group. This 11-beta hydroxy of dexamethasone and prednisolone has been shown in receptor ligand crystals to interact with Asn564. Asn-564 is very important for ligand binding and even more for target gene activation and transcription factor repression [35]. Glucocorticoid receptor interactions with glucocorticoids: evaluation by molecular modeling and functional analysis of glucocorticoid receptor mutants). We hypothesize that, although vamorolone binds to the glucorticoid receptor, the GR-ligand complex does not forma dimerize and is thus not an efficient GR-mediated activator. Further, that this important difference is largely because of the structural differences in the C ring of vamorolone.

Taken together, the data from these first-in-human Phase 1 clinical trials of vamorolone suggest a significant improvement of safety profiles relative to existing pharmacological glucocorticoid drugs. Using multiple serum biomarkers bridged to later clinical safety concerns, we have shown that vamorolone has lost key safety concerns regarding bone turnover, insulin resistance, and immunosuppression. Adrenal suppression was seen at high doses (9.0 and 20.0 mg/kg/day), but this is ~1% of the potency of traditional glucocorticoids.

We did not study aspects of efficacy (e.g. serum cytokines) in these Phase 1 studies, as the volunteers were healthy adults, and did not have a pro-inflammatory state. First-in-patient studies in 4 to < 7 years DMD boys are currently underway and include a 2 weeks MAD study design similar to the Phase 1 MAD study presented here (Phase 2a), and a 6 months Phase 2a extension study for dose finding. Doses planned were chosen based on the pre-clinical and Phase 1 data, and are 0.25, 0.75, 2.0, and 6.0 mg/kg/day, where the 4% suspension formulation is ingested with a glass of full fat milk (food effect). It is important to note that the loss of side effects enables higher dosing of vamorolone relative to standard glucocorticoid drugs. This may improve efficacy relative to prednisone and deflazacort. Phase 2 trials of vamorolone in DMD are planned to go to 10-times prednisone dose. There are additional upsides regarding possible improved efficacy of vamorolone relative to glucocorticoids. Vamorolone is a mineralocorticoid antagonist similar in potency to eplerenone, and this may improve both cardiac and skeletal muscle function in DMD [20,36,37].

A key difference in the molecular mechanism of action of vamorolone, a dissociative steroidal drug, vs. traditional glucocorticoid drugs, likely lies in the dimerization of ligand/receptor complexes. Vamorolone binds both the glucocorticoid receptor and mineralocorticoid receptors with high affinity and shows drug-dependent translocation of the cytoplasmic receptor to the nucleus, very similar to traditional glucocorticoids [14,15,19]. The activity of vamorolone is retained with GRdim (dimerization defective mutants) [15]. The reduced ability of vamorolone to dimerize ligand/receptor complexes leads to loss of the GRE-mediated transcriptional activity increasingly associated with side effects profiles, while also changing the mineralocorticoid receptor activity from an agonist (glucocorticoids) to an antagonist (vamorolone). Thus, vamorolone dissociates not only GR-dependent safety and efficacy features, but also MR-dependent features as well.

## Supplementary Material

supplement

## Figures and Tables

**Table 1:** Summary of pharmacokinetic parameters for vamorolone after oral administration of single 0.1, 0.3, 1, 3, 8, and 20 mg/kg doses to healthy subjects under fasted conditions.

Parameter*	Dose					
	
	0.1 mg/kg	0.3 mg/kg	1 mg/kg	3 mg/kg	8 mg/kg	20 mg/kg
Cmax (ng/mL)	13.1 (12.8) (6)	50.8 (16.5) (6)	122 (32.8) (6)	305 (24.4) (6)	718 (42.5) (6)	1,648 (16.7) (6)
Tmax (h)	1.50 (6)	1.50 (6)	1.75 (6)	1.75 (6)	1.78 (6)	1.50 (6)
	[1.50–2.03]	[1.00–3.00]	[1.00–3.00]	[1.00–2.00]	[1.00–2.00]	[1.00–3.03]
AUC(0-t) (hr × ng/mL)	41.9 (16.8) (6)	161 (15.9) (6)	486 (19.7) (6)	1,577 (20.7) (6)	3,997 (55.0) (6)	8,545 (29.5) (6)
AUC(inf) (hr × ng/mL)	49.5 (12.5) (6)	170 (16.5) (6)	500 (19.2) (6)	1,600 (20.3) (6)	3,602 (60.2) (4)	8,653 (37.0) (4)
λz (1/h)	0.4060 (12.5) (6)	0.4325 (17.8) (6)	0.3828 (17.9) (6)	0.2773 (16.3) (6)	0.2136 (40.9) (4)	0.1629 (25.2) (4)
t½ (h)	1.71 (12.5) (6)	1.60 (17.8) (6)	1.81 (17.9) (6)	2.50 (16.3) (6)	3.25 (40.9) (4)	4.26 (25.2) (4)
CL/F						
(L/h/kg)	2.02 (12.5) (6)	1.76 (16.5) (6)	2.00 (19.2) (6)	1.88 (20.3) (6)	2.22 (60.2) (4)	2.31 (37.0) (4)
(L/h)	168 (20.8) (6)	142 (14.4) (6)	165 (12.0) (6)	152 (18.5) (6)	196 (57.6) (4)	180 (29.8) (4)
Vz/F						
(L/kg)	4.98 (6.14) (6)	4.07 (20.5) (6)	5.22 (18.5) (6)	6.76 (28.7) (6)	10.4 (61.8) (4)	14.2 (37.2) (4)
(L)	415 (17.4) (6)	329 (19.6) (6)	432 (22.8) (6)	550 (28.7) (6)	919 (63.1) (4)	1,107 (34.6) (4)

* Geometric mean (%CV) (N) except Tmax for which the median (N) [Range] is reported.

**Table 2:** Summary of pharmacokinetic parameters for vamorolone after oral administration of single 8 mg/kg doses to healthy subjects under fed and fasted conditions.

Parameter*	8 mg/kg		Ratio†
		
	Fasted	Fed	
Cmax (ng/mL)	718 (42.5) (6)	1,817 (31.4) (6)	2.53
Tmax (h)	1.78 (6)	4.00 (6)	
	[1.00–2.00]	[2.00–6.00]	
AUC(0-t) (hr × ng/mL)	3,997 (55.0) (6)	10,139 (25.1) (6)	2.54
AUC(inf) (hr × ng/mL)	3,602 (60.2) (4)	10,170 (24.9) (6)	2.82
λz (1/h)	0.2136 (40.9) (4)	0.2950 (18.9) (6)	
t½ (h)	3.25 (40.9) (4)	2.35 (18.9) (6)	
CL/F			
(L/h/kg)	2.22 (60.2) (4)	0.79 (24.9) (6)	
(L/h)	196 (57.6) (4)	66.7 (28.4) (6)	
Vz/F			
(L/kg)	10.4 (61.8) (4)	2.67 (23.4) (6)	
(L)	919 (63.1) (4)	226 (29.2) (6)	

* Geometric mean (%CV) (N) except Tmax for which the median (N) [Range] is reported.

† Ratio of the geometric means.

## Abbreviations

11BHSD: 11β-hydroxysteroid dehydrogenase
ACTH: adrenocorticotropic hormone
AE: adverse event
ALT: alanine aminotransferase
AUC: area under the curve
BMI: body mass index
CBG: cortisol binding globulin
CL/F: apparent total clearance of the drug from plasma after oral administration
CTX1: C-terminal telopeptide of collagen 1
ECG: electrocardiogram
GR: glucocorticoid receptor
GRdim: dimerization defective glucocorticoid receptor
GRE: glucocorticoid response element
ICH: International Council on Harmonisation
IP: Investigational product
MAD: Multiple ascending dose
MTD: Maximum tolerated dose
NCATS: National Center for Advancing Translational Sciences
NIH: National Institutes of Health
NOAEL: No observed adverse event level
PI: Principal investigator
PK: Pharmacokinetics
QD: quaque die (once a day)
SAD: Single ascending dose
SAE: Severe adverse event
SRC: Safety Review Committee
TEAE: Treatment-emergent adverse events
ULN: Upper limit of normal
