# Supplementary material for: Phase 1 trial of vamorolone, a first-in-class steroid, shows improvements in side effects via biomarkers bridged to clinical outcomes
Source: Steroids. Author manuscript; Available in PMC 2018 Sep 13. (PMC6136660; doi:10.1016/j.steroids.2018.02.010)
Supplement: supplement [file NIHMS965660-supplement.pdf]

**Supplementary Materials:**

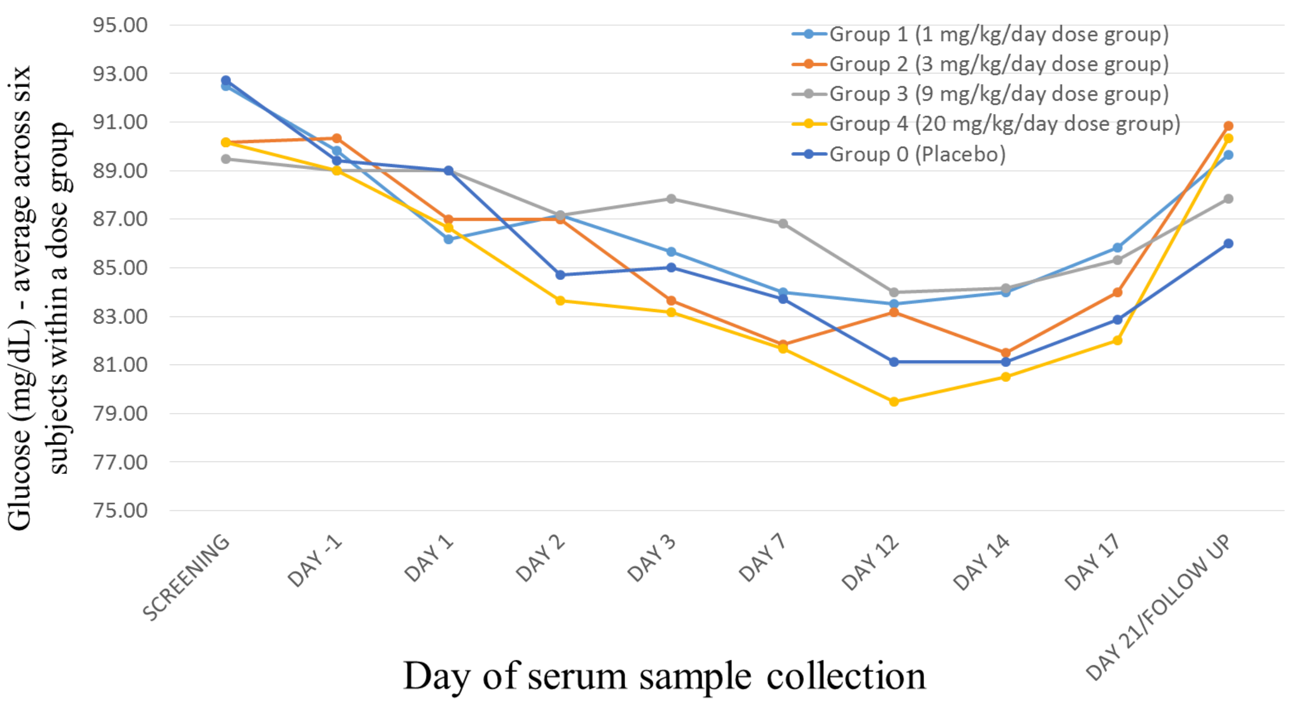


**Supplemental Figure 1. Glucose measures of vamorolone-treated adult volunteers are not significantly different from placebo.** Fasting blood glucose levels were measured on the mornings of the indicated days, with vamorolone or placebo treatment on days 1 – 14. Days 14-21 are recovery time points. Placebo subjects showed a decline over time in the Phase 1 in-patient unit, possibly due to diet, but these differences were similar to all vamorolone-treated groups. This data shows that vamorolone does not cause insulin resistance as seen with glucocorticoids.

**Prednisone treatment**

**Single dose** **7 days daily dosing**

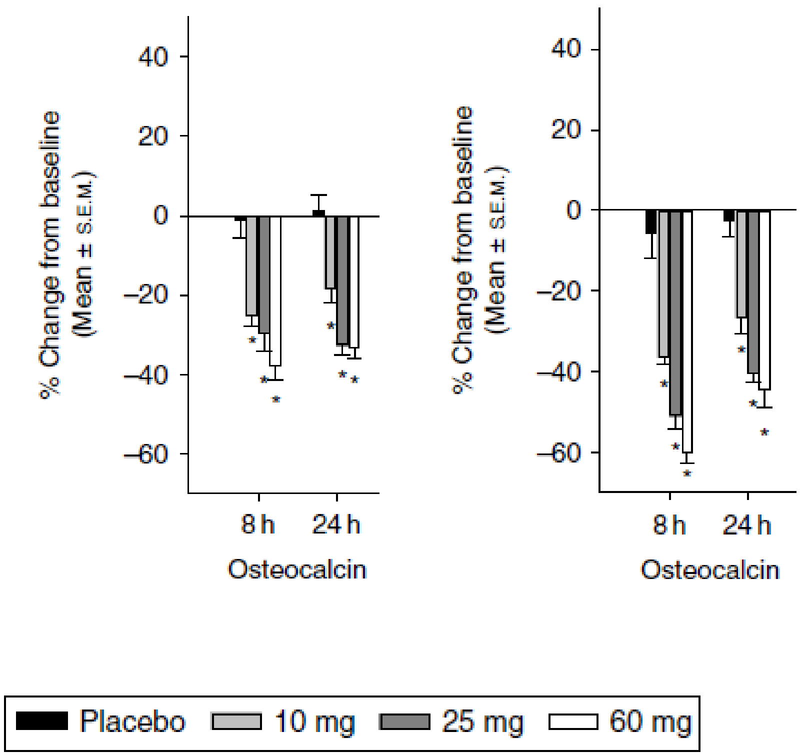


**Equivalencies** **0.125 mg/kg** **0.31 mg/kg** **0.75 mg/kg**

**Supplemental Figure 2. Serum levels of a biomarker of bone formation, osteocalcin, are significantly decreased by 0.125 mg/kg of prednisone (taken from Kauh et al. 2013) (***Error! Bookmark not defined.***).** A 10 mg dose (equivalent to ~0.125 mg/kg) of prednisone shows significant declines in blood levels of osteocalcin both 8 hours after a single dose, and after 7 days of daily dosing. In contrast, vamorolone showed no declines through 20 mg/kg/day. Each prednisone dosed group was compared to placebo using a non-parametric test and reported p-values are adjusted for multiple comparisons; * indicates a statistically significant difference from placebo at the p≤0.05 level.

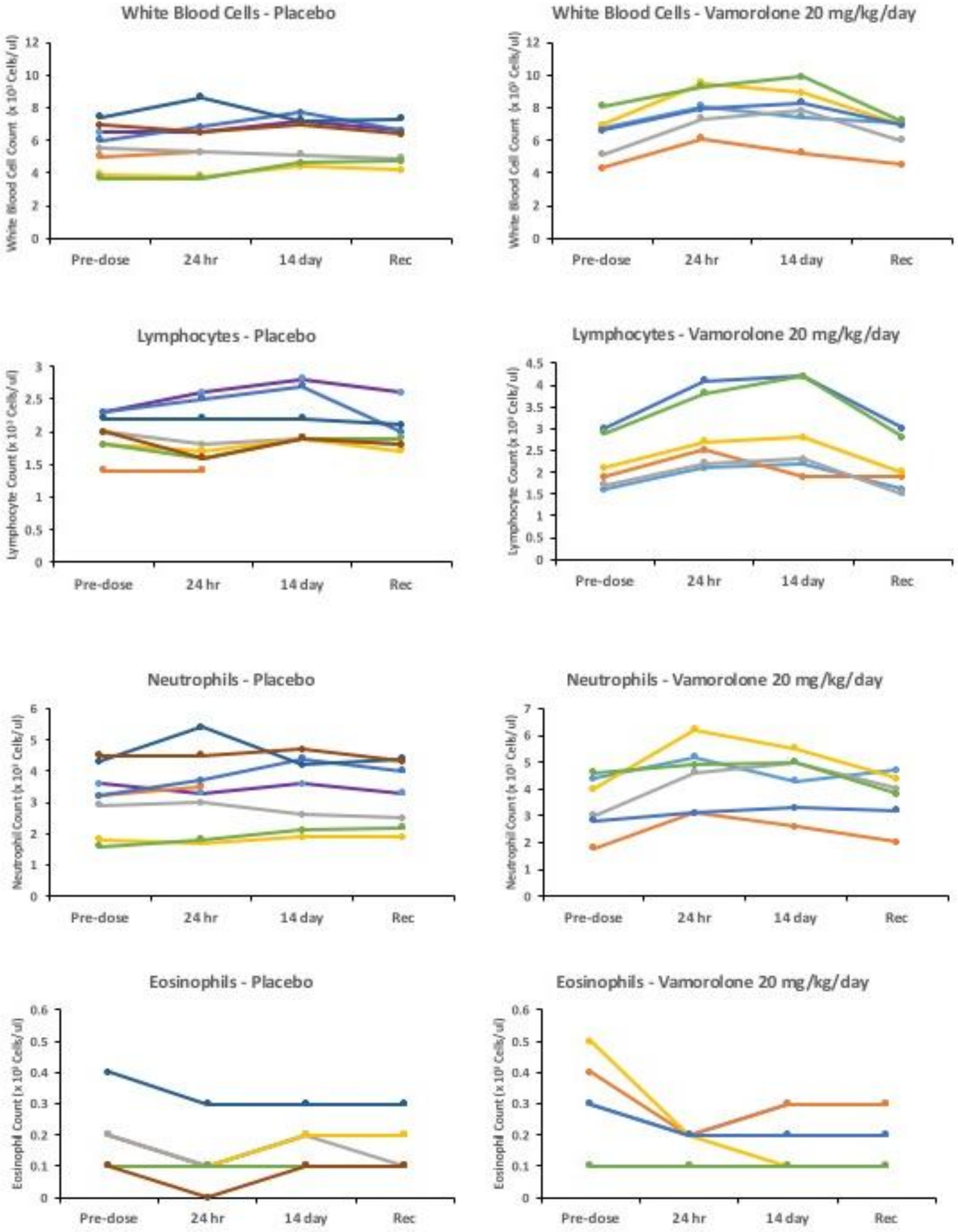


**Supplemental Figure 3. Differential blood counts are not significantly different from placebo.** White blood cells and subsets (lymphocytes, neutrophils, eosinophils) were measured at screening, day -1, pre-dose on days 1-14, day 17, and at follow-up (recovery). Day 1 (pre-dose), Day 2 (24 hr after first dose), Day 14 and follow-up (recovery) time points are graphed

below.  Dosing was halted on Day 9 for one patient in the 20 mg/kg/day cohort (orange line) and one patient in the placebo cohort (purple line).

**Supplemental Tables:**

**All data shown mean ± SEM**

**Supplemental Table 1. Blood glucose measures: Single ascending dose study.**

| Dose | Time Point (mg/dL) | | | | | | |
|---|---|---|---|---|---|---|---|
| | Screening[a] | Day -1[b] | <2 hours predose | 24 Hours Post Dose | 48 Hours Post Dose | 72 Hours Post Dose | Follow-up Visit |
| Placebo | 97 ± 2 | 92 ± 1 | 95 ± 1 | 91 ± 2 | 89 ± 2 | 90 ± 1 | 94 ± 2 |
| 0.1 mg/kg | 94 ± 4 | 99 ± 3 | 95 ± 3 | 95 ± 4 | 95 ± 3 | 94 ± 4 | 98 ± 4 |
| 0.3 mg/kg | 93 ± 2 | 91 ± 2 | 93 ± 2 | 91 ± 2 | 90 ± 2 | 91 ± 1 | 88 ± 4 |
| 1.0 mg/kg | 92 ± 3 | 92 ± 3 | 93 ± 3 | 86 ± 3 | 88 ± 3 | 86 ± 3 | 93 ± 2 |
| 3.0 mg/kg | 96 ± 5 | 89 ± 3 | 92 ± 4 | 93 ± 3 | 89 ± 4 | 88 ± 3 | 91 ± 5 |
| 8.0 mg/kg | 89 ± 4 | 90 ± 5 | 86 ± 3 | 91 ± 4 | 87 ± 3 | 89 ± 4 | 94 ± 4 |
| 20.0 mg/kg | 93 ± 3 | 94 ± 3 | 97 ± 4 | 91 ± 2 | 86 ± 2 | 88 ± 2 | 89 ± 3 |

**Supplemental Table 2. Blood glucose measures: Multiple ascending dose study.**

| Dose | Time Point (md/dL) | | | | | | | | | |
|---|---|---|---|---|---|---|---|---|---|---|
| | Screening[a] | Day -1[b] | Day 1[c] | Day 2[c] | Day 3[c] | Day 7[c] | Day 12[c] | Day 14[c] | Day 17[d] | Day 21[e] |
| Placebo | 93 ± 2 | 90 ± 1 | 90 ± 2 | 85 ± 1 | 85 ± 1 | 85 ± 1 | 85 ± 4 | 81 ± 1 | 83 ± 1 | 86 ± 2 |
| 1.0 mg/kg | 92 ± 1 | 90 ± 2 | 86 ± 1 | 87 ± 2 | 86 ± 2 | 84 ± 2 | 83 ± 1 | 84 ± 1 | 86 ± 1 | 90 ± 3 |
| 3.0 mg/kg | 90.1 ± 1.5 | 90 ± 1 | 87 ± 1 | 87 ± 1 | 84 ± 2 | 82 ± 1 | 83.1 ± 1.4 | 81 ± 2 | 84 ± 1 | 91 ± 2 |
| 9.0 mg/kg | 89 ± 3 | 89 ± 1 | 89 ± 2 | 87 ± 1 | 88 ± 2 | 87 ± 2 | 84 ± 2 | 84 ± 2 | 85 ± 2 | 88 ± 2 |
| 20.0 mg/kg | 90 ± 3 | 89 ± 1 | 87 ± 1 | 84 ± 1 | 83 ± 1 | 82 ± 1 | 80 ± 2 | 80 ± 2 | 81 ± 2 | 91 ± 3 |

[a] Up to 30 days prior to randomization (day 1)
[b] Up to 24 hours prior to first dose
[c] Predose (within 2 hours prior to dosing)
[d] 3 days after last dose (Day 14)
[e] Follow-up visit

**Supplemental Table 3. Osteocalcin: Multiple ascending dose**

| Dose | Osteocalcin: Time Point (ng/ml) | | | | | |
|---|---|---|---|---|---|---|
| | Day 1 (predose)[a] | Day 1[b] | Day 14 (predose)[a] | Day 14[b] | Day 15[c] | Day 17[d] |
| Placebo | 46.3 ± 9.7 | 40.1 ± 7.4 | 42.7 ± 8.8 | 34.0 ± 6.7 | 40.8 ± 7.9 | 38.8 ± 6.2 |
| 1.0 mg/kg | 33.2 ± 11.5 | 27.4 ± 9.3 | 30.3 ± 9.7 | 21.0 ± 7.8 | 28.8 ± 9.3 | 25.9 ± 8.0 |
| 3.0 mg/kg | 13.7 ± 11.6 | 13.6 ± 11.5 | 17.1 ± 13.3 | 13.0 ± 11.0 | 16.1 ± 12.5 | 13.9 ± 9.5 |
| 9.0 mg/kg | 32.9 ± 8.6 | 20.4 ± 4.7 | 24.0 ± 6.4 | 18.7 ± 4.3 | 26.7 ± 5.2 | 33.5 ± 7.2 |
| 20.0 mg/kg | 31.5 ± 6.7 | 20.2 ± 4.6 | 32.8 ± 7.6 | 17.0 ± 3.7 | 27.8 ± 5.9 | 34.6 ± 6.1 |

**Supplemental Table 4. CTX1: Multiple ascending dose**

| Dose | CTX1: Time Point (ng/ml) | | | | | |
|---|---|---|---|---|---|---|
| | Day 1 (predose)[a] | Day 1[b] | Day 14 (predose)[a] | Day 14[b] | Day 15[c] | Day 17[d] |
| Placebo | 0.66 ± 0.12 | 0.28 ± 0.05 | 0.81 ± 0.21 | 0.30 ± 0.07 | 0.88 ± 0.22 | 0.84 ± 0.22 |
| 1.0 mg/kg | 0.50 ± 0.11 | 0.22 ± 0.02 | 0.58 ± 0.12 | 0.22 ± 0.01 | 0.68 ± 0.16 | 0.55 ± 0.09 |
| 3.0 mg/kg | 0.31 ± 0.05 | 0.18 ± 0.00 | 0.48 ± 0.13 | 0.18 ± 0.00 | 0.48 ± 0.10 | 0.46 ± 0.12 |
| 9.0 mg/kg | 0.59 ± 0.11 | 0.19 ± 0.006 | 0.73 ± 0.14 | 0.23 ± 0.03 | 0.77 ± 0.13 | 0.60 ± 0.10 |
| 20.0 mg/kg | 0.70 ± 0.14 | 0.22 ± 0.02 | 0.88 ± 0.12 | 0.29 ± 0.06 | 0.92 ± 0.14 | 0.76 ± 0.10 |

[a] Up to 30 days prior to randomization (day 1)
[b] Up to 24 hours prior to first dose
[c] Predose (within 2 hours prior to dosing)
[d] 3 days after last dose (Day 14)
[e] Follow-up visit

**Supplemental Table 5. White blood cells counts: Multiple ascending dose study**

| Dose | Time Point | | | | | | | | | |
|---|---|---|---|---|---|---|---|---|---|---|
| | Screening[a] | Day -1[b] | Day 1[c] | Day 2[c] | Day 3[c] | Day 7[c] | Day 12[c] | Day 14[c] | Day 17[d] | Day 21[e] |
| Placebo | 5.8 ± 1.6 | 6.0 ± 1.2 | 5.6 ± 1.4 | 5.8 ± 1.6 | 6.2 ± 1.6 | 5.8 ± 1.2 | 6.4 ± 1.6 | 6.2 ± 1.4 | 5.8 ± 1.2 | 6.9 ± 2.9 |
| 1.0 mg/kg | 5.9 ± 2.2 | 5.5 ± 0.8 | 5.2 ± 1.0 | 5.3 ± 0.9 | 5.4 ± 0.8 | 5.9 ± 0.7 | 6.1 ± 0.9 | 5.9 ± 0.9 | 5.7 ± 0.6 | 5.5 ± 0.7 |
| 3.0 mg/kg | 6.2 ± 2.9 | 6.1 ± 1.9 | 5.8 ± 2.0 | 5.9 ± 1.5 | 6.1 ± 2.2 | 6.3 ± 2.0 | 6.0 ± 2.0 | 5.8 ± 1.8 | 6.6 ± 1.6 | 6.3 ± 1.6 |
| 9.0 mg/kg | 5.2 ± 1.3 | 5.5 ± 1.1 | 5.2 ± 1.1 | 6.7 ± 1.2 | 6.8 ± 1.0 | 6.6 ± 1.3 | 6.8 ± 0.9 | 6.8 ± 1.3 | 5.5 ± 0.7 | 5.2 ± 1.1 |
| 20.0 mg/kg | 6.1 ± 1.1 | 7.3 ± 1.1 | 6.3 ± 1.4 | 8.0 ± 1.3 | 7.9 ± 0.7 | 8.2 ± 1.0 | 8.3 ± 0.5 | 8.5 ± 0.4 | 6.8 ± 0.2 | 6.8 ± 0.4 |

**Supplemental Table 6. Neutrophil cell counts: Multiple ascending dose**

| Dose | Time Point | | | | | | | | | |
|---|---|---|---|---|---|---|---|---|---|---|
| | Screening[a] | Day -1[b] | Day 1[c] | Day 2[c] | Day 3[c] | Day 7[c] | Day 12[c] | Day 14[c] | Day 17[d] | Day 21[e] |
| Placebo | 3.5 ± 1.3 | 3.3 ± 0.8 | 3.1 ± 1.0 | 3.4 ± 1.2 | 3.6 ± 1.2 | 3.4 ± 1.0 | 3.5 ± 1.1 | 3.4 ± 1.2 | 3.2 ± 1.0 | 4.2 ± 2.7 |
| 1.0 mg/kg | 3.6 ± 2.0 | 2.8 ± 0.9 | 2.8 ± 1.0 | 3.0 ± 0.9 | 2.8 ± 0.8 | 3.1 ± 0.9 | 3.2 ± 1.0 | 3.0 ± 1.0 | 3.0 ± 0.8 | 3.0 ± 0.6 |
| 3.0 mg/kg | 3.5 ± 1.8 | 3.5 ± 1.4 | 3.4 ± 1.3 | 3.4 ± 1.2 | 3.4 ± 1.4 | 3.3 ± 0.9 | 3.2 ± 1.1 | 3.0 ± 1.1 | 4.0 ± 1.1 | 3.9 ± 1.4 |
| 9.0 mg/kg | 3.0 ± 1.0 | 2.9 ± 0.6 | 2.9 ± 0.9 | 3.7 ± 1.1 | 3.6 ± 0.7 | 3.4 ± 1.0 | 3.5 ± 0.9 | 3.6 ± 1.0 | 3.1 ± 0.7 | 2.9 ± 1.0 |
| 20.0 mg/kg | 3.5 ± 1.2 | 3.9 ± 1.1 | 3.4 ± 1.1 | 4.5 ± 1.2 | 4.3 ± 1.2 | 4.4 ± 0.7 | 4.4 ± 0.4 | 4.6 ± 0.4 | 4.0 ± 0.3 | 4.0 ± 0.3 |

**Supplemental Table 7. Lymphocytes: Multiple ascending dose**

| Dose | Time Point | | | | | | | | | |
|---|---|---|---|---|---|---|---|---|---|---|
| | Screening[a] | Day -1[b] | Day 1[c] | Day 2[c] | Day 3[c] | Day 7[c] | Day 12[c] | Day 14[c] | Day 17[d] | Day 21[e] |
| Placebo | 1.8 ± 0.4 | 2.1 ± 0.5 | 2.0 ± 0.3 | 1.9 ± 0.4 | 2.0 ± 0.5 | 1.9 ± 0.3 | 2.3 ± 0.6 | 2.2 ± 0.4 | 2.0 ± 0.3 | 2.0 ± 0.5 |
| 1.0 mg/kg | 1.8 ± 0.5 | 2.1 ± 0.5 | 1.9 ± 0.4 | 1.8 ± 0.3 | 2.1 ± 0.2 | 2.1 ± 0.3 | 2.3 ± 0.3 | 2.3 ± 0.2 | 2.1 ± 0.4 | 2.0 ± 0.2 |
| 3.0 mg/kg | 2.0 ± 1.0 | 1.9 ± 0.6 | 1.8 ± 0.6 | 2.0 ± 0.4 | 2.1 ± 0.7 | 2.4 ± 0.8 | 2.1 ± 0.7 | 2.2 ± 0.6 | 2.0 ± 0.5 | 1.8 ± 0.6 |
| 9.0 mg/kg | 1.7 ± 0.5 | 2.1 ± 0.7 | 1.8 ± 0.4 | 2.5 ± 0.7 | 2.7 ± 0.7 | 2.6 ± 0.6 | 2.8 ± 0.6 | 2.6 ± 0.6 | 1.9 ± 0.5 | 1.8 ± 0.7 |
| 20.0 mg/kg | 2.0 ± 0.5 | 2.6 ± 0.5 | 2.2 ± 0.6 | 2.9 ± 0.8 | 2.9 ± 0.7 | 3.2 ± 0.9 | 3.2 ± 0.4 | 3.1 ± 0.4 | 2.2 ± 0.3 | 2.2 ± 0.3 |

**Supplemental Table 8. Eosinophils: Multiple ascending dose**

| Dose | Time Point | | | | | | | | | |
|---|---|---|---|---|---|---|---|---|---|---|
| | Screening[a] | Day -1[b] | Day 1[c] | Day 2[c] | Day 3[c] | Day 7[c] | Day 12[c] | Day 14[c] | Day 17[d] | Day 21[e] |
| Placebo | 0.15 ± 0.08 | 0.20 ± 0.13 | 0.18 ± 0.10 | 0.11 ± 0.08 | 0.13 ± 0.07 | 0.14 ± 0.07 | 0.16 ± 0.05 | 0.17 ± 0.08 | 0.16 ± 0.08 | 0.16 ± 0.11 |
| 1.0 mg/kg | 0.13 ± 0.08 | 0.18 ± 0.10 | 0.17 ± 0.08 | 0.17 ± 0.08 | 0.17 ± 0.08 | 0.20 ± 0.14 | 0.20 ± 0.09 | 0.18 ± 0.08 | 0.17 ± 0.08 | 0.15 ± 0.10 |
| 3.0 mg/kg | 0.18 ± 0.08 | 0.20 ± 0.06 | 0.23 ± 0.08 | 0.23 ± 0.08 | 0.20 ± 0.06 | 0.18 ± 0.08 | 0.18 ± 0.04 | 0.20 ± 0.06 | 0.17 ± 0.05 | 0.18 ± 0.04 |
| 9.0 mg/kg | 0.10 ± 0.00 | 0.12 ± 0.04 | 0.10 ± 0.00 | 0.13 ± 0.05 | 0.10 ± 0.00 | 0.10 ±0.00 | 0.12 ± 0.04 | 0.13 ± 0.05 | 0.08 ± 0.04 | 0.12 ± 0.04 |
| 20.0 mg/kg | 0.25 ± 0.14 | 0.32 ± 0.16 | 0.28 ± 0.16 | 0.17 ± 0.05 | 0.15 ± 0.08 | 0.15 ± 0.08 | 0.12 ± 0.02 | 0.16 ± 0.04 | 0.16 ± 0.04 | 0.26 ± 0.02 |

**Supplemental Table 9. Crystallographic data of vamorolone**

| | Vamorolone |
|---|---|
| Crystal data | |
| Chemical formula | $C_{22}H_{28}O_4$ |
| $M_r$ | 356.44 |
| Crystal system, space group | Orthorhombic, $P2_12_12_1$ |
| Temperature (K) | 110 |
| *a*, *b*, *c* (Å) | 8.18746 (9), 18.0748 (2), 37.5595 (4) |
| *V* ($Å^3$) | 5558.31 (10) |
| *Z* | 12 |
| Radiation type | Cu *Kα* |
| □ ($mm^{-1}$) | 0.69 |
| Crystal size (mm) | 0.51 × 0.38 × 0.31 |
| | |
| Data collection | |
| Diffractometer | SuperNova, Dual, Cu at zero, Atlas |
| Absorption correction | Analytical<br>*CrysAlis PRO*, Agilent Technologies, Version 1.171.36.32 (release 02-08-2013 CrysAlis171 .NET) (compiled Aug 2 2013,16:46:58). Analytical numeric absorption correction using a multifaceted crystal model based on expressions derived by R.C. Clark & J.S. Reid. (Clark, R. C. & Reid, J. S. (1995). Acta Cryst. A51, 887-897) |
| $T_{min}$, $T_{max}$ | 0.774, 0.844 |
| No. of measured, independent and observed [$I > 2\sigma(I)$] | 36042, 10890, 10330 |

| | |
|---|---|
| reflections | |
| $R_{int}$ | 0.017 |
| $(\sin \theta/\lambda)_{max}$ (Å$^{-1}$) | 0.616 |
| | |
| Refinement | |
| $R[F^2 > 2\sigma(F^2)]$, $wR(F^2)$, $S$ | 0.030, 0.089, 1.06 |
| No. of reflections | 10890 |
| No. of parameters | 719 |
| H-atom treatment | H-atom parameters constrained |
| $\Delta\rho_{max}$, $\Delta\rho_{min}$ (e Å$^{-3}$) | 0.24, -0.13 |
| Absolute structure | Flack x determined using 4305 quotients [(I+)-(I-)]/[(I+)+(I-)] (Parsons, Flack and Wagner, Acta Cryst. B69 (2013) 249-259). |
| Absolute structure parameter | -0.02 (3) |
